# Walkability parameters, active transportation and objective physical activity: moderating and mediating effects of motor vehicle ownership in a cross-sectional study

**DOI:** 10.1186/1479-5868-9-123

**Published:** 2012-10-05

**Authors:** Ulf Eriksson, Daniel Arvidsson, Klaus Gebel, Henrik Ohlsson, Kristina Sundquist

**Affiliations:** 1 Center for Primary Health Care Research, Lund University, Malmö, Sweden; 2 Stanford Prevention Research Center, Stanford University School of Medicine, Stanford, California, USA; 3 City Futures Research Centre, Faculty of the Built Environment, University of New South Wales, Sydney, Australia; 4 School of Education, University of Newcastle, Newcastle, Australia; 5 Prevention Research Collaboration, Sydney School of Public Health, University of Sydney, Sydney, Australia

**Keywords:** Accelerometer, Neighborhood walkability, Geographic information system, Mediator, Moderator

## Abstract

**Background:**

Neighborhood walkability has been associated with physical activity in several studies. However, as environmental correlates of physical activity may be context specific, walkability parameters need to be investigated separately in various countries and contexts. Furthermore, the mechanisms by which walkability affects physical activity have been less investigated. Based on previous research, we hypothesized that vehicle ownership is a potential mediator. We investigated the associations between walkability parameters and physical activity, and the mediating and moderating effects of vehicle ownership on these associations in a large sample of Swedish adults.

**Methods:**

Residential density, street connectivity and land use mix were assessed within polygon-based network buffers (using Geographic Information Systems) for 2,178 men and women. Time spent in moderate to vigorous physical activity was assessed by accelerometers, and walking and cycling for transportation were assessed by the International Physical Activity Questionnaire. Associations were examined by linear regression and adjusted for socio-demographic characteristics. The product of coefficients approach was used to investigate the mediating effect of vehicle ownership.

**Results:**

Residential density and land use mix, but not street connectivity, were significantly associated with time spent in moderate to vigorous physical activity and walking for transportation. Cycling for transportation was not associated with any of the walkability parameters. Vehicle ownership mediated a significant proportion of the association between the walkability parameters and physical activity outcomes. For residential density, vehicle ownership mediated 25% of the association with moderate to vigorous physical activity and 20% of the association with the amount of walking for transportation. For land use mix, the corresponding proportions were 34% and 14%. Vehicle ownership did not moderate any of the associations between the walkability parameters and physical activity outcomes.

**Conclusions:**

Residential density and land use mix were associated with time spent in moderate to vigorous physical activity and walking for transportation. Vehicle ownership was a mediator but not a moderator of these associations. The present findings may be useful for policy makers and city planners when designing neighborhoods that promote physical activity.

## Background

The interest in environmental determinants of physical activity behavior has been rapidly increasing over the past few years. Ecological models are often used as a basis to describe the multi-component influence of individual factors, the social environment and the physical environment on physical activity [1-3]. Objective measures of neighborhood walkability, a construct commonly including residential density, street connectivity and land use mix, have been associated with physical activity in several studies [4-7]. For example, participants from the Swedish Neighborhood and Physical Activity (SNAP) study living in highly walkable neighborhoods spent more time in moderate to vigorous physical activity (MVPA) and reported more walking for leisure and walking for transportation compared to participants living in less walkable neighborhoods [6]. That study investigated the association between an overall walkability index and physical activity, but it did not stratify the analyses by the different components of walkability (residential density, street connectivity, and land use mix). As associations between the environment and physical activity are context-specific, it is of interest to investigate the effects of the separate walkability parameters on physical activity under various conditions. To our knowledge, no previous study has investigated the association between objectively assessed walkability parameters and physical activity in a northern European context.

Previous cross-sectional studies have found negative associations between neighborhood walkability and motor vehicle ownership (further referred to as vehicle ownership) [8] and vehicle miles traveled [9,10]. This implies that dense, well connected areas with diverse land use could support a less car-dependent living. Vehicle ownership and vehicle use are, in turn, negatively associated with physical activity [8,11]. We hypothesize that vehicle ownership may lie in the causal pathway between neighborhood walkability and physical activity. To our knowledge, no previous study has investigated the hypothesized mediating effect of vehicle ownership on the association between objectively assessed walkability parameters and physical activity.

Vehicle ownership may also moderate associations between the physical environment and physical activity. A recent study found a positive association between convenience of bus services and physical activity in non-drivers, but not in drivers [12]. Furthermore, a Belgian study found significantly more steps per day among participants with a preference for passive transport living in highly walkable neighborhoods compared to participants with the same preference living in less walkable neighborhoods. This difference was not found in participants with a preference for active transport, but their number of steps per day was generally higher [13]. To examine potential moderators of the relationship between the environment and physical activity is the most frequently suggested direction for future research outlined in reviews on environment and physical activity research [14].

The first aim of this study was to investigate the associations between objectively assessed residential density, street connectivity, and land use mix and physical activity outcomes, i.e. time spent in MVPA, walking for transportation and cycling for transportation. The second aim was to investigate the hypothesized pathway between walkability parameters and physical activity through vehicle ownership using mediation analysis. The third aim was to test whether the associations between the walkability parameters and physical activity are modified by vehicle ownership.

## Methods

### Study design

The present study uses cross-sectional data from the Swedish Neighborhood and Physical Activity (SNAP) study, collected between November 2008 and November 2009 in Stockholm. Stockholm municipality covers 188 square kilometers and has a population of about 850,000 inhabitants. It is the central city in a metropolitan area with about 2.1 million inhabitants. Participants for the SNAP study were recruited from neighborhoods differing in walkability assessed by Geographic Information Systems (GIS) and neighborhood-level income as described below. A full description of the design of the SNAP study has been provided elsewhere [6].

The city of Stockholm is divided into 408 administrative areas, with about 2,000 people living in each area. These areas were used as a basis for the calculations of the neighborhood-level variables. Neighborhood walkability was calculated as an index comprising of the sum of z-scores of residential density, street connectivity and land use mix. Some previous studies included retail floor area ratio as one of the components of their walkability measure and weighted street connectivity by 2 [15]. In this study, where information of retail floor area ratio was not available, street connectivity was weighted by 1.5. Administrative areas within the first to fourth deciles of walkability index were considered to be less walkable and the seventh to tenth deciles were considered to be highly walkable. Neighborhood-level income was calculated as the median family income, taking the age and numbers of family members into account. The second to fourth deciles of neighborhood-level income were considered as low neighborhood-level income and the seventh to ninth deciles were considered as high. The first and tenth deciles were excluded to avoid outliers in neighborhood-level income. A total of 127 administrative areas were classified into the following four categories: high walkability/high income, high walkability/low income, low walkability/high income or low walkability/low income. Administrative areas in the high walkability/high income category located in the city center were rather small. Therefore, some areas in this category were merged to create study neighborhoods. A total of 32 neighborhoods, eight in each of the four categories, were included in the study.

### Study sample

A total of 8,000 individuals (250 from each neighborhood) aged 20 to 65 were randomly selected. Of these, 6,089 had a listed landline or cell phone number and were included in the recruitment procedure. A week after an information letter was sent to the individuals, a telemarketing company (Markör AB, Örebro, Sweden) called them to recruit participants and to answer any questions that they might have. To be included in the study, participants had to meet three inclusion criteria: 1) being able to read and write in Swedish, 2) having no serious impaired ability to walk and 3) having lived in the neighborhood for at least three months. Of the 4,747 individuals who were reached by phone, 4,369 met the inclusion criteria and 3,226 agreed to participate in the study. Recruitment was done concurrently in all 32 neighborhoods and data were collected throughout the year except for weeks 50 to 2 and weeks 25 to 33, corresponding to the Christmas and summer holidays. Lists of enrolled participants were delivered to the research group on a weekly basis. A package containing an accelerometer, an accelerometer logbook, a questionnaire and a pre-paid return envelope was sent to the participants. After participation, the participants received a pedometer, movie tickets or lottery tickets at a value of about 100 SEK (1 SEK=0.11 EUR or 0.15 USD). A total of 2,178 participants had complete GIS, accelerometer and self-report data and were included in the analyses.

### Neighborhood walkability parameters

Neighborhood walkability parameters were objectively measured using GIS. Each participant’s residential address was geo-coded and 1,000-meter polygon-based network buffers were created around the residences using the Network Analyst extension in ArcGIS/ArcInfo 9.2 (ESRI Inc., Redlands, California, USA). Network buffers, compared to predefined administrative areas or circular buffers, may better reflect a “true” area of exposure. Polygon-based network buffers (further referred to as buffers) were created by following the road network including bicycle paths and footpaths in all possible directions for 1,000 meters from the residence and then drawing a line to connect the endpoints, thus creating a polygon shaped area (a buffer) surrounding the residence. Buffers of 1,000 meters have often been used in previous research as studies have found that it is a distance many people are willing to walk in their daily life [16]. Detailed network data were delivered by the City Planning Administration in Stockholm and included the road network as well as bicycle paths and footpaths. Highways were excluded from the data.

**Residential density** was based on data obtained from Statistics Sweden and calculated as the number of residential units (in ten thousands) per square kilometer. **Street connectivity** was based on the same network data as when creating the buffer zones. That is, the data was delivered by the City Planning Administration in Stockholm, and it included the road network, bicycle paths and footpaths. Highways were excluded from the calculations. Bicycle paths and footpaths that run parallel with roads often result in multiple intersections within one “true” intersection. Therefore, a buffering procedure was employed where two or more intersections closer to each other than 10 meters were counted as one. Street connectivity was calculated as the number of intersections per square kilometer. **Land use mix** was calculated as the evenness in distribution between five categories of land use: 1) retail/service, 2) entertainment/physical activity, 3) institutional/healthcare, 4) office/workplace, and 5) dwellings. Categories 1 to 4 were based on data delivered by Teleadress, a company founded when the government-owned telecom sector was privatized. The Teleadress database is updated continuously and it includes businesses and services with a registered phone number, as well as those who actively have provided information about their business. The fifth category was based on data obtained from the City Planning Administration in Stockholm. The level of land use mix was based on point data and calculated by the Herfindahl-Hirschman Index (HHI). The HHI is calculated by summing the squared proportions of each land use category (HHI= p_1_^2^ + p_2_^2^… + p_5_^2^). A high HHI indicates a low level of land use mix. In this study, however, the HHI-values were reversed (multiplied by −1) to facilitate interpretation of results (making a higher HHI correspond to a higher level of land use mix). We then divided the HHI-values by 10,000. This was done in order to make the unit in the explanatory variable and the regression coefficients easier to interpret, representing a meaningful difference in the neighborhood environment. For example, one increase in the unit of residential density used in the analyses (10,000 dwellings per square kilometer), corresponded to a shift from the lowest density to a mid-range density in this sample. The ranges of the explanatory variables are shown in Table 1.

**Table 1:** Descriptive statistics on the 2,178 individuals included in the study

	**Median or percent**	**Interquartile range**	**Min; max**
Residential density (residential units x 10^-4^/km^2^)	0.23	0.14; 0.43	0.06; 1.77
Street connectivity (intersections/km^2^)	86.4	73.4; 102.1	30.5; 155.3
Land use mix (HHI x 10^-4^ x (−1))^a^	−0.76	−0.86; -0.36	−0.98; -0.24
Age:			
20–30	11%		
31–40	21%		
41–50	28%		
51–66	40%		
Gender ( females)	55%		
Income:			
Low	19%		
Middle	56%		
High	25%		
Marital status (married/cohabiting)	75%		
Vehicle ownership:			
0	18%		
1	48%		
≥2	34%		
Moderate to vigorous physical activity (min/day)	41.3	27.1; 57.9	0.1; 183.7
Walking for active transportation (min/week)	125	30; 300	0; 1260
Cycling for active transportation (min/week)^b^	0	0; 20	0; 1260

^a^In this study, a higher Herfindahl-Hirschman Index correspond to a higher level of land use mix.

^b^Observations collected between April and October (n=906).

### Time spent in moderate to vigorous physical activity

The time spent in moderate to vigorous physical activity was objectively assessed with Actigraph GT1M accelerometers (ActiGraph, Pensacola, FL, USA). Actigraph GT1M is uni-axial and registers acceleration in the vertical plane. The accelerometers were set to sum the physical activity (counts) within 60-second periods (epoch) and participants were asked to wear them during all waking hours for seven consecutive days and to only remove them when engaging in water activities. Participants were given the opportunity to choose accelerometer placement on the hip or lower back to increase compliance. A study comparing accelerometer placement on the hip or lower back under free-living conditions found no significant effect of the placement on the estimation of time spent in moderate to vigorous physical activity [17]. Four text messages were sent to participants during the seven-day period to further increase compliance. Non-wear time was defined as ≥60 continuous minutes of zero counts. A minimum of ten hours of wear time was required to constitute a valid day and participants with six or more valid days were included in the analysis. Variance analysis of our own data was performed to determine the number of valid days required to capture habitual physical activity [18]. Time spent in MVPA was defined using Freedson’s cut-off as ≥1,952 counts per minute [19].

### Walking and cycling for active transportation

The amount of walking for transportation and cycling for transportation in minutes per week was assessed by the long self-administered version of the International Physical Activity Questionnaire (IPAQ). The IPAQ has shown good reliability and fair to moderate validity when using accelerometers as the criterion [20]. The frequency and duration of walking and cycling for transport purposes during the past seven days are reported. Data were cleaned and scored according to the official IPAQ scoring protocol (sites.google.com/site/theipaq/scoring-protocol). Due to the low proportions of participants reporting cycling during November-March (7-13%), the analyses on cycling for transportation only included observations collected between April and October where 20-32% of participants reported cycling for transportation during the past seven days (n=906).

### Vehicle ownership

The numbers of vehicles in the household were based on information from the study questionnaire in which participants were asked: “How many roadworthy motor vehicles do you have in your household?” Vehicle ownership was categorized into three levels: no vehicle, one vehicle and two or more vehicles.

### Socio-demographic information

Socio-demographic data were based on self-report. Age was categorized into four levels: 20–30 years, 31–40 years, 41–50 years, and 51–66 years. Marital status was dichotomized into either married/cohabiting with a partner or living without a partner. Income was calculated by dividing the gross family income by number of people living in the household, with children/adolescents under the age of 18 being given a consumption weight of 0.5. Income was then categorized into three levels: low (<150,000 SEK/year), middle (150,000-349,999 SEK/year) and high (≥350,000 SEK/year). One SEK equals about 0.11 EUR or 0.15 USD (August 2012).

### Statistical analysis

**Figure 1:**
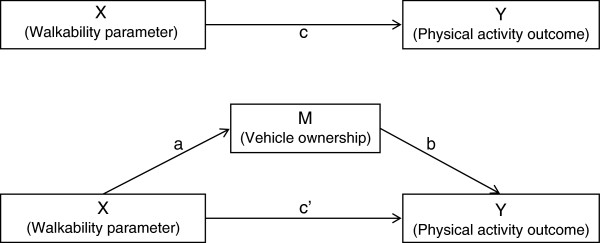
**a and b The associations between X and Y without (Figure**1**a) and with a mediator (Figure**1**b).** X represents the explanatory variables; residential density, street connectivity or land use mix. Y represents the outcome variables; MVPA, walking for active transportation or cycling for active transportation. M represents the potential mediator; vehicle ownership

We investigated the association between three different walkability parameters (residential density, street connectivity and land use mix) and three different outcomes (MVPA, walking for transportation and cycling for transportation). Further, we investigated whether these associations were mediated and/or moderated by vehicle ownership. Figure 1a illustrates a potential direct effect of X on Y, while Figure 1b illustrates the mediation design where the product of a and b (a*b) is the potentially mediating effect of M on the association between X and Y. Walking for transportation and cycling for transportation were investigated both as dichotomous variables (yes/no) and as log transformed variables (with individuals that had a value higher than 0).

Linear regression was used to investigate the associations between the walkability parameters and the physical activity outcomes. To investigate the mediating effect of vehicle ownership on these associations we used an approach described by Preacher and Hayes [21]. This approach uses bootstrapping, a nonparametric resampling procedure, to generate confidence intervals for the indirect effect. We also calculated the proportion mediated, by dividing a*b with c. To check the robustness of our results, we also performed non-parametric analyzes using PROC GENMOD in SAS v. 9.2 (SAS Institute, Cary, NC, USA) with the identity link and specified the variance to be binomial as well as using ordinary logistic regression. The mediated proportions in these control results were very similar to the results shown in the tables. For all outcomes we also investigated the potential interaction between vehicle ownership and the different walkability parameters.

### Models

In all models we first investigated the association between the different walkability parameters and the physical activity outcomes. Thereafter we included the socio-demographic characteristics in the models in order to investigate if the association was confounded by individual characteristics (full model).

### Non-response analysis

A telephone-based non-response analysis of 205 persons, randomly selected from those who were reached by phone but declined participation, was performed. There was no difference in income between participants and non-participants but the proportion of females was slightly higher among participants and participants were slightly older than non-participants.

### Ethics

Ethical approval for this study was granted by the Ethics Committee of Karolinska Institutet, Stockholm. Written informed consent was obtained from all participants.

## Results

Participants had a median of 86.4 intersections per square kilometer (Interquartile Range, IQR=73.4-102.1; Range=30.5-155.3), 2,300 dwellings per square kilometer (IQR=1,400-4,300; Range=600-17,700) and an HHI of 7,600 (IQR 3,600-8,600; Range=2,400-9,800) within their buffer zones, as shown in Table 1. As described in the methods section, residential density was divided by 10,000 and HHI was divided by 10,000 and multiplied by −1 to facilitate interpretation of results. The study sample consisted of 55% females, 75% were married/cohabiting, 68% were over the age of 40 and 19% were in the low income group. The median value of time spent in MPVA was 41.3 minutes per day (IQR=27.1-57.9) and the median for walking for transportation was 125 minutes per week (IQR=30-300). Individuals participating in the study between April and October had a median of 0 minutes per week of cycling for transportation (IQR=0-20).

### Walkability parameters and MVPA

Table 2 shows the associations between the different walkability parameters, vehicle ownership and MVPA as well as the mediating effects of vehicle ownership. A paths illustrate the associations between walkability parameters and vehicle ownership; b paths illustrate the associations between vehicle ownership and MVPA (note that the walkability parameters are not included in the b paths); c paths illustrate the associations between walkability parameters and MVPA, and c’ paths represent c paths adjusted for vehicle ownership.

**Table 2:** Walkability parameters, vehicle ownership and MVPA. Numbers represent regression coefficients (95% confidence intervals)

	**a paths**	**b paths**^**a**^	**c paths**	**c’ paths**	Indirect effects (a paths*b paths)	**Proportion mediated**
Residential density	−0.53	−3.05	6.81	5.20	1.61	24%
	(−0.60; -0.46)	(−4.50; -1.59)	(4.35; 9.27)	(2.63; 7.77)	(0.81; 2.48)	
Residential density	−0.49	−2.95	5.86	4.42	1.44	25%
(Full model^b^)	(−0.56; -0.42)	(−4.45; -1.46)	(3.37; 8.35)	(1.84; 7.01)	(0.69; 2.31)	
Street connectivity	n/a	n/a	0.02 (−0.02; 0.07)	n/a	n/a	n/a
Street connectivity	n/a	n/a	n/a	n/a	n/a	n/a
(Full model^b^)						
Land use mix	−1.00	−3.07	10.30	7.24	3.06	30%
	(−1.11; -0.88)	(−4.55; -1.60)	(6.25; 14.35)	(2.95; 11.53)	(1.56; 4.67)	
Land use mix	−0.90	−3.11	8.13	5.33	2.80	34%
(Full model^b^)	(−1.02; -0.78)	(−4.62; -1.60)	(3.94; 12.32)	(0.94; 9.72)	(1.32; 4.25)	

a paths: Associations between walkability parameters and vehicle ownership.

b paths: Associations between vehicle ownership and MVPA (minutes/day).

c paths: Associations between walkability parameters and MVPA (minutes/day).

c’ paths: c paths adjusted for vehicle ownership.

^a^b paths are not based on the walkability parameters.

^b^Adjusted for age, gender, income and marital status.

n/a: Not applicable (as no significant association was found between the walkability parameter and the physical activity outcome).

The results of the regression analyses show that residential density and land use mix was positively associated with time spent in MVPA. An increase of residential density of 10,000 dwellings per square kilometer was associated with 6.8 (CI=4.4-9.3) more minutes per day of MVPA. This association remained significant when adjusting for age, gender, marital status and income (full model). For land use mix, an increase of the HHI by 10,000 was associated with 10.3 (CI=6.3-14.4) more minutes per day of MPVA and this association remained significant in the full model (c paths). No significant association was found between street connectivity and time spent in MVPA. There were negative associations between residential density as well as land use mix and vehicle ownership (a paths). There were also negative associations between vehicle ownership and time spent in MVPA in both models (b paths). Vehicle ownership mediated 25% of the association between residential density and time spent in MVPA in the full model and this mediating effect was statistically significant. For land use mix, the corresponding figure was 34%.

### Walkability parameters and walking for transportation

Table 3 shows the logistic regression analyses and Table 4 shows the linear regression analyses of the association between the walkability parameters, vehicle ownership and walking for transportation as well as the mediating effects of vehicle ownership. Residential density and land use mix were significantly and positively associated with reporting walking for transportation (yes/no) and with the amount of walking for transportation (log transformed minutes per week) in the full models. Street connectivity was significantly and positively associated with walking for transportation in the linear regression analysis (c paths). There were negative associations between vehicle ownership and walking for transportation in both the logistic and the linear regression analyses (b paths). Vehicle ownership mediated 20% of both the logistic and the linear associations between residential density and walking for transportation in the full models. For land use mix, the corresponding figures were 22% and 14% for the logistic and linear associations, respectively, and these mediating effects were statistically significant.

**Table 3:** Walkability parameters, vehicle ownership and walking for transportation (yes/no). Numbers represent regression coefficients (95% CI)

	**a paths**	**b paths**^**a**^	**c paths**	**c’ paths**	Indirect effects (a paths*b paths)	**Proportion mediated**
						
Residential density	−0.53	−0.06	0.14	0.11	0.03	22%
	(−0.60; -0.46)	(−0.08; -0.03)	(0.10; 0.18)	(0.07; 0.15)	(0.02; 0.04)	
Residential density	−0.49	−0.05	0.13	0.10	0.03	23%
(Full model^b^)	(−0.56; -0.42)	(−0.08; -0.03)	(0.09; 0.17)	(0.06; 0.15)	(0.01; 0.04)	
Street connectivity	n/a	n/a	0.0003	n/a	n/a	n/a
			(−0.000; 0.001)			
Street connectivity	n/a	n/a	n/a	n/a	n/a	n/a
(Full model^b^)						
Land use mix	−1.00	−0.06	0.23	0.18	0.06	26%
	(−1.11; -0.88)	(−0.08; -0.03)	(0.16; 0.30)	(0.10; 0.25)	(0.03; 0.08)	
Land use mix	−0.90	−0.05	0.21	0.16	0.05	24%
(Full model^b^)	(−1.02; -0.78)	(−0.08; -0.03)	(0.14; 0.28)	(0.09; 0.24)	(0.03; 0.07)	

a paths: Associations between walkability parameters and vehicle ownership.

b paths: Associations between vehicle ownership and walking for active transportation (dichotomous, yes/no).

c paths: Associations between walkability parameters and walking for active transportation (dichotomous, yes/no).

c’ paths: c paths adjusted for vehicle ownership.

^a^b paths are not based on the walkability parameters.

^b^Adjusted for age, gender, income and marital status.

n/a: Not applicable (as no significant association was found between the walkability parameter and the physical activity outcome).

**Table 4:** Walkability parameters, vehicle ownership and walking for transportation (amount*)

	**a paths**	**b paths**^**a**^	**c paths**	**c’ paths**	Indirect effects (a paths*b paths)	**Proportion mediated**
Residential density	−0.49	−0.11	0.26	0.21	0.05	19%
	(−0.56; -0.41)	(−0.18; -0.03)	(0.14; 0.38)	(0.08; 0.33)	(0.02; 0.09)	
Residential density	−0.45	−0.11	0.28	0.24	0.05	18%
(Full model^b^)	(−0.53; -0.37)	(−0.18; -0.03)	(0.16; 0.40)	(0.12; 0.36)	(0.02; 0.08)	
Street connectivity	−0.002	−0.137	0.003	0.002	0.0003	11%
	(−0.004; -0.001)	(−0.207; -0.067)	(0.000; 0.005)	(0.000; 0.004)	(0.0001; 0.0007)	
Street connectivity	n/a	n/a	0.002	0.002	n/a	n/a
(Full model^b^)			(0.000; 0.005)	(−0.000; 0.004)		
Land use mix	−0.98	−0.09	0.53	0.44	0.09	17%
	(−1.12; -0.86)	(−0.16; -0.02)	(0.33; 0.74)	(0.23; 0.66)	(0.02; 0.16)	
Land use mix	−0.87	−0.09	0.58	0.50	0.08	14%
(Full model^b^)	(−1.00; -0.74)	(−0.17; -0.02)	(0.37; 0.79)	(0.27; 0.73)	(0.02; 0.15)	

Numbers represent regression coefficients (95% CI).

*Only individuals that have reported some walking are included and values are log-transformed.

a paths: Associations between walkability parameters and vehicle ownership.

b paths: Associations between vehicle ownership and walking for active transportation (log-transformed min/week).

c paths: Associations between walkability parameters and walking for active transportation (log-transformed min/week).

c’ paths: c paths adjusted for vehicle ownership.

^a^b paths are not based on the walkability parameters.

^b^Adjusted for age, gender, income and marital status.

n/a: Not applicable (as no significant association was found between the walkability parameter and the physical activity outcome).

### Walkability parameters and cycling for transportation (not shown in tables)

None of the walkability parameters were associated with reporting cycling for transportation (yes/no) or with the amount of cycling for transportation (log transformed minutes per week).

### Effect modification by vehicle ownership

Table 5 shows the results from the interaction tests between the walkability parameters and vehicle ownership. There was no significant effect modification by vehicle ownership on any of the associations between the walkability parameters and the physical activity outcomes.

**Table 5:** Interaction analysis between walkability parameters and vehicle ownership

	**Residential density**	**Street connectivity**	**Land use mix**
MVPA	0.806	0.112	0.589
Walking for transportation (0/1)	0.266	0.809	0.918
Walking for transportation (log)	0.889	0.575	0.953
Cycling for transportation (0/1)	0.091	0.647	0.124
Cycling for transportation (log)	0.429	0.547	0.555

Values are p-values for the interaction term.

## Discussion

The aim of this study was to investigate the associations between three walkability parameters (residential density, street connectivity and land use mix) and physical activity and to analyze the mediating and moderating effects of vehicle ownership on these associations. The results showed that residential density and land use mix, objectively assessed within 1,000 meter network buffers around participants’ residences, are positively associated with time spent in MVPA and walking for transportation. This is in line with previous research investigating objectively assessed residential density and land use mix as separate measures [22] or when incorporating these measures in indexes of overall walkability [5-7].

Street connectivity was weakly associated with the amount of walking for transportation, but it was not associated with any of the other physical activity outcomes in this study. The lack of associations between street connectivity and physical activity outcomes is in contrast to some earlier findings from other studies. For example, Frank et al. found street connectivity to be significantly associated with moderate physical activity [22]. The non-significant association between street connectivity and physical activity in this study could be explained by a relatively high level of connectivity. The median number of intersections per square kilometer was 87 in this Swedish study, compared to a mean of 37 intersections per square kilometer found by Frank and colleagues in North America [22]. The lack of association between street connectivity and physical activity found in this study is, however, in line with the conclusions of a review by Saelens and Handy on environmental correlates of walking [23]. They found that while residential density and land use mix were consistently associated with walking for transportation, the findings for street connectivity were more equivocal.

We did not find any significant associations between walkability parameters and cycling for transportation. Even though we included the cycling infrastructure in our data, walkability was developed as a measure of supportive environments for walking and not cycling. Furthermore, a 1000-meter buffer may be too small to capture the area of exposure for cyclists. A study conducted in Stockholm investigating route distances in 110 street-recruited bicycle commuters found a mean commuting distance of 6.7 and 8.0 kilometers for women and men, respectively [24]. However, even smaller buffer zones (450m) have been used in previous research on environmental correlates of cycling for transportation [25]. Furthermore, it may be more common for cyclists to commute from residences in low walkable neighborhoods to workplaces in dense inner city areas than the opposite scenario, in order to avoid traffic congestions and parking problems. This would attenuate an association between neighborhood walkability and cycling for transportation. Future studies could explore this hypothesis using measures of walkability parameters around participants’ workplaces as well as their homes.

Previous studies have found positive associations between neighborhood walkability and active transport (walking + cycling) [10,26]. Other studies have examined the association between wakability and cycling for transportation alone. For example, participants in the Belgian Environmental Physical Activity Study living in highly walkable neighborhoods (walkability assessed within administrative areas) reported 40 minutes more cycling for transportation per week compared to participants living in less walkable neighborhoods [7]. Results from a study by Winters and colleagues showed positive associations between objectively assessed population density, street connectivity and land use mix and cycling for transportation [25]. Furthermore, Titze et al. found a positive association between perceived street connectivity and cycling for transportation [27].

Vehicle ownership mediated a statistically significant proportion of all the significant associations between walkability parameters and physical activity outcomes. For example, 34% of the association between land use mix and time spent in MVPA were mediated by vehicle ownership. To our knowledge, no previous studies have investigated vehicle ownership as a mediator between objectively assessed walkability parameters and physical activity outcomes. Therefore, our results are hard to compare with the currently available knowledge base. However, our results are in line with the findings of a study by Sehatzadeh et al. in which fewer vehicles were owned by households in walkable environments and where the number of vehicles in the household was negatively associated with frequency of walking [8]. This is also supported by results from a longitudinal study by Mumford and colleagues, where participants reported more walking and less automobile use after moving to a community with a high land use mix [28].

We did not find any significant effect modification by vehicle ownership on the associations between walkability parameters and physical activity outcomes. Participants living in dense areas with a mixed land use spent more time in MVPA and reported more walking for transportation compared to participants living in areas with lower residential density and land use mix, regardless of vehicle ownership. This is in contrast to some previous findings where vehicle ownership, or similar vehicle-related measures, moderated the relationship between the environment and physical activity. For example, driving status modified the association between convenience of bus services and physical activity in a Japanese study [12] and preference for passive transport modified the association between walkability and numbers of steps per day in a Belgian setting [13]. However, the present study and the studies by Kamada et al. and Van Dyck et al. used different explanatory as well as outcome measures. For example, preference for passive transport may have a different influence on the association between walkability parameters and physical activity compared to vehicle ownership.

This study has some limitations that should be considered. It is a cross-sectional study and therefore causality cannot be determined. Self-report measures of walking and cycling for transportation may include bias due to social desirability and difficulties to recall activities during the past seven days. Accelerometers, on the other hand, do not suffer from these biases and provide an objective measure of physical activity on a moderate to vigorous intensity level. Strengths of this study also include the large number of participants (n=2,178) and the objective measures of walkability parameters using network buffers. The network buffers were based on detailed network data, including the road network as well as bicycle paths and footpaths. This provides a more relevant area of exposure for cyclists and pedestrians compared to network buffers based solely on the road network. Finally, participants were recruited from neighborhoods with a wide range of walkability and neighborhood-level SES, which is an additional strength.

## Conclusions

The present study showed a positive association between two out of three walkability parameters (residential density and land use mix but not street connectivity) and time spent in moderate to vigorous physical activity and walking for transportation. Significant proportions of these associations were mediated by vehicle ownership. Interaction tests suggested that residential density and land use mix are favorable for physical activity regardless of vehicle ownership status. Our findings may be useful for policy makers and city planners when designing physical activity promoting neighborhoods. We welcome future evaluations of the parameters incorporated in environmental indices, such as the walkability index, in different countries.

## Abbreviations

SNAP: the Swedish Neighborhood and Physical Activity study; GIS: Geographic Information Systems; MVPA: moderate to vigorous physical activity.

## Competing interests

None of the authors have any conflicts of interest to declare.

## Authors’ contributions

All authors contributed to the conception and design of the study. UE and KS contributed to the acquisition of data. UE and HO performed the statistical analysis. All authors contributed to interpretation of data, revision of the manuscript for important intellectual content and final approval of the manuscript.
